# Consistent diversity loss but divergent functional responses of freshwater biofilms to salinity and nutrient enrichment across climatic regions

**DOI:** 10.1098/rspb.2026.1161

**Published:** 2026-10-21

**Authors:** Rose Fuggle, Mariana Mayer-Pinto, Miguel G. Matias, Ezequiel M. Marzinelli

**Affiliations:** ^1^ The University of Sydney, New South Wales, Australia; ^2^ University of New South Wales, Sydney, New South Wales, Australia; ^3^ Sydney Institute of Marine Science, Mosman, New South Wales, Australia; ^4^ Museo Nacional de Ciencias Naturales Madrid, Community of Madrid, Spain

**Keywords:** biofilms, freshwater ecosystems, freshwater management, freshwater salinization, multiple stressors, nutrient enrichment, stressor interactions, bacterial communities, benthic microbial communities

## Abstract

Freshwater salinization and nutrient enrichment are intensifying globally. Despite their widespread co-occurrence, the generality of their combined effects across regions remains unclear, limiting identification of appropriate policy scales. Using outdoor freshwater ponds established >10 years ago, replicated across inland, coastal and mountain regions, we examined independent and interactive effects of elevated salinity (0, +5 and +15 g NaCl l⁻¹) and nutrient enrichment (10 : 1 N : P additions) on benthic biofilms. Salinity was the dominant driver of community structure and function. Nutrient enrichment increased green algae abundances after 30 days but did not affect biofilm function or bacterial structure. Across all regions, bacterial richness and Shannon's diversity index declined consistently with increasing salinity after 30 days and bacterial community structures differed among all salinity levels by 60 days. Responses of phototrophic groups and biofilm functions, however, were region-specific. In the inland region, high salinity increased biofilm biomass, gross primary productivity (GPP), net primary productivity (NPP) and abundances of diatoms and cyanobacteria; in the coastal region, GPP and NPP declined under high salinity with no change to phototroph abundances; while neither was affected in the mountain region. In sum, taxonomic changes in biofilm bacterial communities under salinization were consistent across regions, while functional changes varied.

## 1. Introduction

Freshwater lakes and ponds are vital ecosystems that sustain biodiversity, drive primary productivity, regulate carbon cycling and provide water resources, yet they are increasingly threatened worldwide [1,2]. Nutrient enrichment has long been recognized as a dominant stressor threatening freshwater biodiversity and ecosystem functions. Emerging stressors, particularly those that co-occur and interact with nutrient enrichment, are of growing concern [2–5]. Freshwater salinization is one of the most pervasive and concerning of such emerging stressors [6–8]. Freshwater salinization is accelerating across a wide range of climatic zones owing to increasing agricultural fertilization and irrigation practices, over-extraction of groundwater, mining, road de-icing using salts, climate-driven alterations to precipitation patterns, salt water intrusion and sea-level rise [6,7]. Rising salinity imposes physiological stress on freshwater organisms by altering osmotic balance and ion toxicity, in turn reducing biodiversity and reshaping communities [9]. Freshwater salinization often co-occurs with nutrient enrichment, as intensive land-use practices including irrigation, fertilization and mining run-off increase salts *and* nutrients in freshwater systems [10]. The few studies that have investigated these combined effects show that they can interactively reshape freshwater community structure and function across various trophic levels [10–13]. However, the extent to which ecological effects of multiple stressors are generalizable and predictable across environmentally distinct regions remains an outstanding knowledge gap [14]. Testing the generality of effects of multiple stressors across distinct climatic regions is critical for determining appropriate scales for policy and management.

Recent experimental research has demonstrated that the functional consequences of freshwater salinization and nutrient enrichment vary significantly depending on temporal context [15]. Both the order of stressor exposure and the number of stressor events significantly influenced the metabolic outcomes for benthic biofilms [15]. When salinity was applied before nutrient enrichment, rates of carbon metabolism were impaired compared to untreated biofilms [15]. When nutrient enrichment preceded salinity, rates of carbon metabolism increased compared to untreated controls [15]. Given these combined stressors’ effects vary with temporal context, a key outstanding question is whether these stressor effects also vary with spatial context.

Most experimental research on the independent and interactive effects of salinity and nutrient enrichment in freshwater systems has relied on laboratory microcosms [10,13] or outdoor mesocosms in a single climatic region [11,12,15]. These experimental systems do not allow for generality in responses across different regions with varying species’ pools and environmental conditions to be tested. Spatially replicated studies using salinity as a single freshwater stressor have demonstrated that salinity effects can be consistent across different environmental contexts [16]. However, meta-analyses across freshwater [14,17], marine [18] and terrestrial [19] systems consistently show that the effects of multiple stressors on community diversity and function vary greatly across studies. The variation among studies may arise because effects of stressors are dependent on local/regional environmental conditions and/or species’ pools [14,20]. Local environmental conditions can drive different higher-order interactions among abiotic factors, e.g. where negative effects of warming and salinity on growth-rates of freshwater bacterial taxa strengthen as pH decreases [21]. Structural traits of communities can also influence stressor effects, e.g. where effects of drought on decomposition rates in soils depend on microbial community composition [22]. However, confounding factors or methodological differences between studies may also explain the variability in stressor effects reported among different studies. Therefore, field experiments replicated across environmentally distinct regions are necessary to reveal the extent to which effects of multiple stressors are genuinely mechanistically context-dependent. This understanding may inform management. For example, where stressor effects are consistent across sites in different regions, it may be appropriate to apply the same management strategies at national or even global scales. Contrastingly, if multiple stressor effects differ significantly across regions, locally tailored approaches will likely be required.

The degree of context-dependency in ecosystem responses to stressors is likely to vary depending on which ecosystem property (community diversity, function) is being measured [12,23]. This is particularly relevant for microbial communities, where high levels of taxonomic diversity within functional groups and the capacity for horizontal gene transfer can often lead to functional redundancy [24]. Additionally, the physiological plasticity of microorganisms is such that important functional changes can occur without changes in community taxonomic structures [12,15]. Therefore, consistent effects of stressors on community structure and diversity across different environments may not translate into consistent functional responses, which may be more context-dependent [24] (but see [25]).

Microbial biofilms, complex communities of bacteria, microalgae, fungi, viruses and protists embedded in an extracellular matrix [26], are foundational to freshwater ecosystem functioning. Biofilms drive primary production and carbon and nutrient cycling, forming the metabolic base that supports higher trophic levels [27]. Increases in salinity typically reduce bacterial growth, biofilm formation and overall abundance, while altering community structure towards more salt-tolerant taxa [28–31]. Salinity can also decrease biomass and photosynthetic yield in biofilm primary producers [9,32,33]. By contrast, nutrient enrichment often stimulates productivity, biofilm thickness and bacterial growth-rates [34–36] while selecting for fast-growing taxa that homogenize communities [37–39]. Nutrient enrichment also drives increases in community respiration, carbon metabolism and extracellular enzyme activity [36,40,41]. When salinity and nutrients co-occur, key biofilm functions such as carbon metabolism can be more strongly impaired than under either stressor individually [12,15].

Here, we assessed the effects of different levels of salinity (0, +5 and +15 g NaCl l⁻¹) and nutrient enrichment (10 : 1 N : P additions), independently and in combination, on benthic biofilm bacterial community structure and whole-community function across three contrasting climatic regions. We used the Iberian Pond Network, a network of semi-natural outdoor freshwater mesocosms with over 10 years of independent succession across inland and coastal Mediterranean regions and a mountain region. This system allows us to test the generality of responses of communities under different environmental conditions and species’ pools [42]. We quantified bacterial community structure, abundances of phototrophic groups (green algae, diatoms and cyanobacteria) and biofilm function (community respiration (CR), net primary productivity (NPP), gross primary productivity (GPP), photosynthetic yield and biomass) after 30 and 60 days. Although nutrient enrichment represents a short-term (‘pulse’) disturbance, our objective was to test whether such pulses have lasting effects that alter biofilm responses to concurrent long-term (‘press’) stressors such as salinity. Consequently, our sampling design is explicitly targeted at detecting longer-term interaction effects. The 30-day timeframe allowed for biofilms to reach the ‘development phase’ of maturation, in which organisms form a relatively taxonomically stable community, embedded in an adherent extracellular matrix [43]. The 60-day sample point allowed us to test for temporal consistency in stressor effects. By focusing on the stabilized biofilm phase, we assess whether prior exposure to a transient nutrient pulse leads to persistent effects that modify biofilm sensitivity to salinity over ecologically relevant timescales.

Benthic biofilms are multi-trophic assemblages composed of heterotrophic bacteria together with phototrophic taxa such as cyanobacteria and eukaryotic algae, and multicellular heterotrophs including fungi and protists. Here, we analysed different response variables (e.g. bacteria, green algae and diatoms) which represent different components of the biofilm. Our 16S rRNA sequencing captures bacterial community structure, while our quantification of green algae, diatom and cyanobacterial abundances provides a broad measure of phototrophic community structure. By contrast, measures of photosynthetic yield and oxygen-flux metrics integrate metabolism across the whole biofilm, including fungi, protists and viruses in addition to bacteria and phototrophs. We expect some functional responses to be directly linked to changes in both bacterial and phototrophic community structures. For example, an increase in cyanobacteria, diatoms and/or green algae could be expected to drive increases in rates of NPP. By contrast, a change in GPP may reflect changes in components of the biofilm community that are not explicitly profiled here, including other algal groups, fungi, protists, other small eukaryotes and/or larger organisms that interact with the biofilm (e.g. grazers). Therefore, our hypotheses address stressor responses on each biofilm component separately.

If salinity and nutrient enrichment do not interact, we hypothesized that [1] moderate (+5 g NaCl l^-1^) and, to a greater extent, high (+15 g NaCl l^-1^) salinity decreases bacterial diversity and richness, shifts bacterial community structures, decreases abundances of phototrophs and decreases rates of biofilm CR, NPP, GPP, photosynthetic efficiency and biomass compared to ambient salinity; [2] nutrient enrichment would decrease bacterial diversity and richness but would increase abundances of phototrophs and increase rates of CR, NPP, GPP, photosynthesis and biomass in biofilms. If salinity and nutrient enrichment interact, we hypothesized that [3] moderate or high salinity and nutrient enrichment in combination would drive stronger structural shifts, declines in bacterial diversity, declines in phototrophic abundance and overall functional declines compared to either stressor individually. We hypothesized that [4] responses in the structure of bacterial communities and in the abundance of phototrophic taxa would be consistent across regions, regardless of whether stressors were applied individually or in combination. This is because of the strong deterministic filtering effects of salinity and nutrient enrichment on freshwater microorganisms. We expected that some of these structural changes would coincide with changes in whole-biofilm functioning. For example, we expected an increase in phototroph abundances to correlate with an increase in photosynthetic rates and NPP, which would be consistent among regions. However, we predicted that other functional responses, such as biomass and GPP, would vary across space. This is because these integrated metrics depend on complex cross-trophic interactions unique to each spatially distinct community. Accordingly, we hypothesized that [5] whole-biofilm functional responses to stressors would vary among regions.

## 2. Methods

### (a) Study area

The Iberian Pond Network [42,44] is a multiregional outdoor mesocosm system built in 2014 to investigate the ecological consequences of freshwater stressors across different bioclimatic regions in complex, semi-natural freshwater communities. At each region, 32 ponds (open outdoor tubs: 1000 l, 0.70 m deep, 1.85 m diameter, placed approx. 3–5 m apart) were initiated by adding locally collected topsoil (100 kg) and well water. The ponds were subsequently colonized by diverse organisms, resulting in established freshwater communities resembling natural small ponds [42,45]. The water columns are dominated by alternating cycles of phytoplankton and zooplankton, while the benthos contains aquatic macrophytes, invertebrates and microorganisms. For this study, we selected three regions, Evora, Porto and Jaca, based on their distinct climatic conditions. Evora is an ‘inland Mediterranean region’ (Csa Köppen Climate Classification System; ‘I’) that has a temperate climate with hot, dry summers (annual mean maximum temperature: 22.5°C, annual mean rainfall: 571.8 mm). Porto is a ‘coastal Mediterranean region’ (Csb Köppen Climate Classification System; ‘C’) with a temperate climate and mild summers (annual mean maximum temperature:19.0°C, annual mean rainfall:1265 mm). Jaca has a temperate oceanic climate (Cfb Köppen Climate Classification System) (annual mean maximum temperature: 18.5°C, annual mean rainfall: 767.5 mm) [46,47]. The site used in this experiment sits above Jaca at an elevation of 1200 m and experiences below-freezing winters (hereafter ‘mountain region’; ‘M’).

### (b) Experimental design

To test for the effects of different levels of salinity and nutrient enrichment, independently and in combination, at each region four ponds (randomly selected, spatially interspersed) were allocated to one of six treatments (*n* = 24 ponds per region): ambient salinity + ambient nutrients (ASAN), ambient salinity + enriched nutrients (ASEN), moderate salinity + ambient nutrients (MSAN), moderate salinity + enriched nutrients (MSEN), high salinity + ambient nutrients (HSAN) or high salinity + enriched nutrients (HSEN). Ambient salinity + ambient nutrients ponds acted as undisturbed controls and received no treatment. Moderate and high salinity ponds were treated by dissolving commercially available salt (sodium chloride, NaCl) (5 g NaCl l^–1^ or 15 g NaCl l^–1^, respectively), in a 20 l bucket with pond water then adding the dissolved salts back into the pond. Nutrient-enriched ponds were treated by dissolving 10 mg N l^–1^ (as sodium nitrate; NaNO_3_; GPR RECTAPUR) and 1 mg of P l^–1^ (as sodium dihydrogen phosphate; NaH_2_PO_4_; GPR RECTAPUR) in a 20 l bucket with pond water then adding the dissolved nutrients back into the pond.

The salinity and nutrient levels in this experiment were selected for their ecological relevance to freshwater salinization and nutrient enrichment. 5 g NaCl l^–1^ (which corresponded to approx. 10 mS cm^–1^; supplementary material, figure S1) is a commonly used experimental salinity level in freshwater salinization experiments reflecting salt concentrations in freshwater systems affected by agricultural irrigation [9]. 15 g NaCl l^–1^ (which corresponded to approx. 30 mS cm^–1^; supplementary material, figure S1) reflects salt concentrations in freshwater ponds undergoing extreme salinization, for example, following road salting for de-icing [48]. For nutrient enrichment, we used a 10× nutrient addition, corresponding to an increase of +10 mg N l^–1^ and +1 mg P l^–1^. Nitrate concentrations of 10 mg l^–1^ reflect the threshold for eutrophication risk and adverse human health impacts set by the US Environmental Protection Agency [49].

### (c) Pond conditions

To quantify the effects of salinity and nutrient treatments on pond water, 30- and 60- days post-treatment, dissolved oxygen (mg O_2_ l^–1^), conductivity (mS cm^–1^) and pH were measured using a HQ40d Multi-Meter (Hach, Australia) with the IntelliCAL luminescent/optical dissolved oxygen probe conductivity and pH probes directly in pond water [15]. Chlorophyll *a* concentration was also quantified using an AquaFluor (Turner Designs) portable fluorometer [46]. Additionally, on day 30, water samples were collected in 100 ml plastic jars to quantify concentrations of nitrate and phosphate using UV-VIS spectrophotometry [46]. Data from each sampling point were normalized and Euclidean distances were calculated among sample pairs. These were analysed using permutational multivariate analysis of variance (PERMANOVA) fitting models described below (§2h) using PRIMER v6 (PRIMER-E, UK). Principal component analysis (PCA) was used to visualize multivariate patterns of water condition.

### (d) Biofilm development and sampling

At each region, 96 ceramic tiles (2 × 2 cm^2^) were randomly allocated to one of 24 plastic trays. Each tray received four tiles. One hour after ponds had received their salinity and nutrient treatments, one tray of tiles was deployed randomly into each of the 24 treatment ponds in each region. Trays were positioned directly on top of the sediment. After 30 and 60 days, one randomly selected tile from each pond was used to quantify bacterial community structure (see §2e below), and a second randomly selected tile was used to quantify abundances of green algae, diatoms and cyanobacteria, rates of CR, NPP, GPP, photosynthetic yield and biomass of the biofilms (see §2g below).

### (e) Community structure

To quantify bacterial community structure, richness and Shannon's diversity in biofilms, at each sampling point (30 and 60 days), one randomly selected tile was removed from each pond of each treatment (*n* = 4 per time point) and gently shaken in the pond water. The entire surface of the tile was then swabbed for 30 s using a sterile cotton swab. Samples were frozen in sterile cryotubes at –20°C in a portable freezer for 24 hours and then stored at –80°C until DNA extraction.

### (f) DNA extraction, sequencing and bioinformatics

DNA was extracted using the DNeasy PowerSoil Pro DNA kit (QIAGEN) as per manufacturer instructions. PCR was performed to amplify the V3-V4 region of the 16S rRNA gene (341-forward primer (CCTACGGGNGGCWGCAG) and 805-reverse primer (GACTACHVGGGTATCTAATCC)) [50]. PCR protocol was adapted from the Illumina library preparation workflow (pre-heating at 95°C for 3 min, followed by 35 cycles of 95°C for 15 s, 55°C for 1 min and 73°C for 30 s). Amplicon sequencing was performed with an Illumina NextSeq P1 sequencer at the Ramaciotti Centre for Genomics.

Raw sequences were processed through the DADA2 package (v 4.3.2) [51] in R (v 4.4.1). Reads were quality-filtered and base pairs with a mean Q score <20 were dropped, resulting in a truncation at 290 bp for both forward and reverse reads [52]. Maximum error rates were calculated and the DADA2 denoising algorithm was used to detect and remove rare amplicon sequence variants (ASVs) and chimaeras. After denoising, paired reads were merged, and an ASV abundance table was constructed. Taxonomic assignment was initially performed using the SILVA reference database (v 138.1) [53] to detect and remove chloroplast, mitochondrial and archaeal sequences (each <1% of total ASVs). A second round of taxonomic assignment was performed using the Genome Taxonomy Database (v 202) [54]. Singletons and ASVs with abundances <0.001% of the total ASV count were also removed, which reduced the ASV count from approximately 70 000 to approximately 8000 while retaining 86.6% of sequences. Relative ASV abundances were normalized using the DeSeq2 variance-stabilizing transformation [55]. ASV richness and Shannon's diversity indices were calculated in the R vegan package [56].

Beta-diversity analyses were performed using square-root transformed normalized ASV abundances to calculate Bray–Curtis dissimilarities among sample-pairs, performed using PRIMER v6 (PRIMER-E, UK). Dissimilarities were analysed using PERMANOVA fitting models described below (see §2h). Non-metric multi-dimensional scaling (nMDS) ordination was used to visualize multivariate patterns of community structure.

### (g) Productivity, photosynthesis and biomass

To measure CR, NPP and GPP, tiles were placed in watertight jars containing 150 ml of water from their source pond. Water was filtered to 20 µm to minimize the contribution of larger waterborne photosynthetic organisms (e.g. phytoplankton) to oxygen production and consumption. Smaller organisms (e.g. waterborne bacteria) may have contributed to these oxygen fluxes, although their contribution is assumed to be much lower than the benthic bacteria given the significantly lower density of waterborne bacteria compared to bacteria in biofilms [57]. Dissolved oxygen concentration was measured pre- and post-2-hour incubations in the dark, and then pre- and post-2-hour incubations in the light using the HQ40d Multi-Meter (Hach, Australia) with the IntelliCAL luminescent/optical dissolved oxygen probe. To calculate GPP, we first quantified CR as the difference between the oxygen concentration at the start and at the end of the dark incubation period. We then calculated NPP as the difference between the oxygen concentration at the end and at the beginning of the light incubation period. GPP rates were calculated by adding CR and NPP [58,59]. CR, NPP and GPP data were not recorded at the mountain site on day 60 owing to equipment failure.

After incubation, tiles were removed from jars and individually dark-adapted for 5 minutes. Maximum photosynthetic quantum yields were then measured using a pulse amplitude modulated fluorometer (WALZ, Germany) [10]. Finally, abundances of green algae, diatoms and cyanobacteria were quantified using a BenthoTorch fluorometer which distinguishes between chlorophyll *a* pigments from major phytoplankton groups based on excitation profiles at 680 nm (BBE, Moldaenke) [60]. The sum of those chlorophyll *a* concentrations was used as a proxy for biofilm biomass [61,62].

### (h) Statistical analyses

To test hypotheses about the effects of salinity (fixed, three levels: AS, MS and HS), nutrients (fixed, crossed, two levels: AN and EN), region (fixed, crossed, three levels: I, C and M) and day (fixed, crossed, two levels: 30 days and 60 days) on conditions in water (conductivity, dissolved oxygen concentration, chlorophyll *a* concentration, pH), and on the structure and function of benthic biofilm communities (Shannon's diversity index on ASVs, bacterial community ASV richness, abundances of green algae, diatoms and cyanobacteria, photosynthetic yield, biomass, CR, NPP and GPP), univariate response variables were analysed via linear mixed effects models using the lmer function from the ‘lme4’ package in R [63]. ‘Pond’ was included as a random factor nested in the interaction between salinity, nutrients and region to account for repeatedly sampling ponds through time. For CR, NPP and GPP, data were not available for the mountain region on day 60 owing to equipment failure, so region had three levels for day 30 data (I, C and M) and two levels for day 60 data (I and C). To test hypotheses about the effects of salinity, nutrients and region (levels as above) on nutrient concentrations in pond water after 30 days, nitrate and phosphate concentrations were analysed via linear models using the lm R function. For all univariate models, *p*-values were calculated using the *F*-statistic with Satterthwaite degrees of freedom in the ‘Anova’ function (type 2) of the R ‘car’ package [64]. Where appropriate, post hoc tests were conducted using the emmeans package in R [65]. Residuals versus fitted values were inspected to check model assumptions and model fit.

For multivariate analyses of water condition (pairwise Euclidean distances on normalized biotic and abiotic water variables listed above), data were separated by day because nitrate and phosphate concentrations were only available for day 30. For multivariate analyses of bacterial community structure (pairwise Bray–Curtis dissimilarities on normalized, relative ASV abundances), models testing the effects of salinity, nutrients, region and day (all levels as listed above), with pond as a random factor, and post hoc tests were run using PERMANOVA in PRIMER v6 (PRIMER-E, UK) [66]. Multivariate dispersions were checked for homogeneity by analysing distances from centroid data using Permdisp in PRIMER [67].

## 3. Results

### (a) Water profiles differed among regions, salinity treatments and nutrient treatments

Conductivity was significantly higher in high salinity ponds compared to ambient and moderate salinity, and significantly higher in moderate salinity ponds compared to ambient salinity, at all regions and through time (salinity × region × day interaction term: *F*_4,52.9_ = 18.50, *p* < 0.001; supplementary material, figure S9 and table S1). pH was significantly higher in ambient salinity ponds compared to high salinity in the mountain and coastal regions, as well as compared to moderate salinity in the coastal region, which was consistent through time (salinity × region × day interaction term: *F*_4,52.5_ = 5.544, *p* = 0.001; supplementary material, figure S1 and table S1). Chlorophyll *a* concentration was significantly higher in high salinity ponds compared to ambient salinity in the inland region, and was significantly higher in ambient salinity ponds compared to moderate salinity in the coastal region (salinity × region interaction term: *F*_4,53.4_ = 3.689, *p* = 0.010; supplementary material, figure S1 and table S1). Dissolved oxygen concentration was significantly higher in high salinity ponds compared to ambient salinity on day 30, while by day 60 this reversed with ambient salinity ponds having significantly higher dissolved oxygen concentration than high salinity (salinity × day interaction term: *F*_2,53.1_ = 6.001, *p* = 0.004; supplementary material, figure S1 and table S1).

Nitrate concentration was significantly higher in nutrient-enriched ponds compared to ambient 30 days after treatment (*F*_1,54_ = 14.229, *p* < 0.001; supplementary material, figure S2 and table S2). Chlorophyll *a* concentration was also significantly higher in nutrient-enriched ponds compared to ambient nutrient ponds in the inland region on day 30 (nutrient × day interaction term: *F*_2,53.3_ = 3.663, *p* = 0.032; supplementary material, figure S1 and table S1). There were no effects of nutrient or salinity treatments on phosphate concentration (supplementary material, figure S2 and table S2).

Environmental conditions differed significantly among all regions despite an interaction with salinity (salinity × region interaction terms: day 30: pseudo-*F*_4,54_ = 1.784, *p* = 0.044; day 60: pseudo-*F*_4,52_ = 5.280, *p* = 0.001; figure 1; supplementary material, figure S1 and table S3 and S4), based on multivariate water profiles (conductivity, chlorophyll *a*, pH, dissolved oxygen concentration, nitrate and phosphate concentrations). Within each region, salinity significantly affected multivariate water profiles, where all salinity levels significantly differed from each other, except for the ambient and moderate salinity treatments in the inland Mediterranean region on day 30 (salinity × region interaction term: D30 pseudo-*F*_4,54_ = 1.784, *p* = 0.044; figure 1; supplementary material, table S3) and ambient and moderate salinity treatments in the mountain region on day 60 (salinity × region interaction term: pseudo-*F*_4,52_ = 5.280, *p* = 0.001; supplementary material, figure S1 and table S2). On day 30, multivariate water profiles were also significantly affected by nutrient enrichment (pseudo-*F*_1,54_ = 5.190, *p* = 0.001; figure 1; supplementary material, table S3), but not day 60 (supplementary material, table S4).

**Figure 1.**
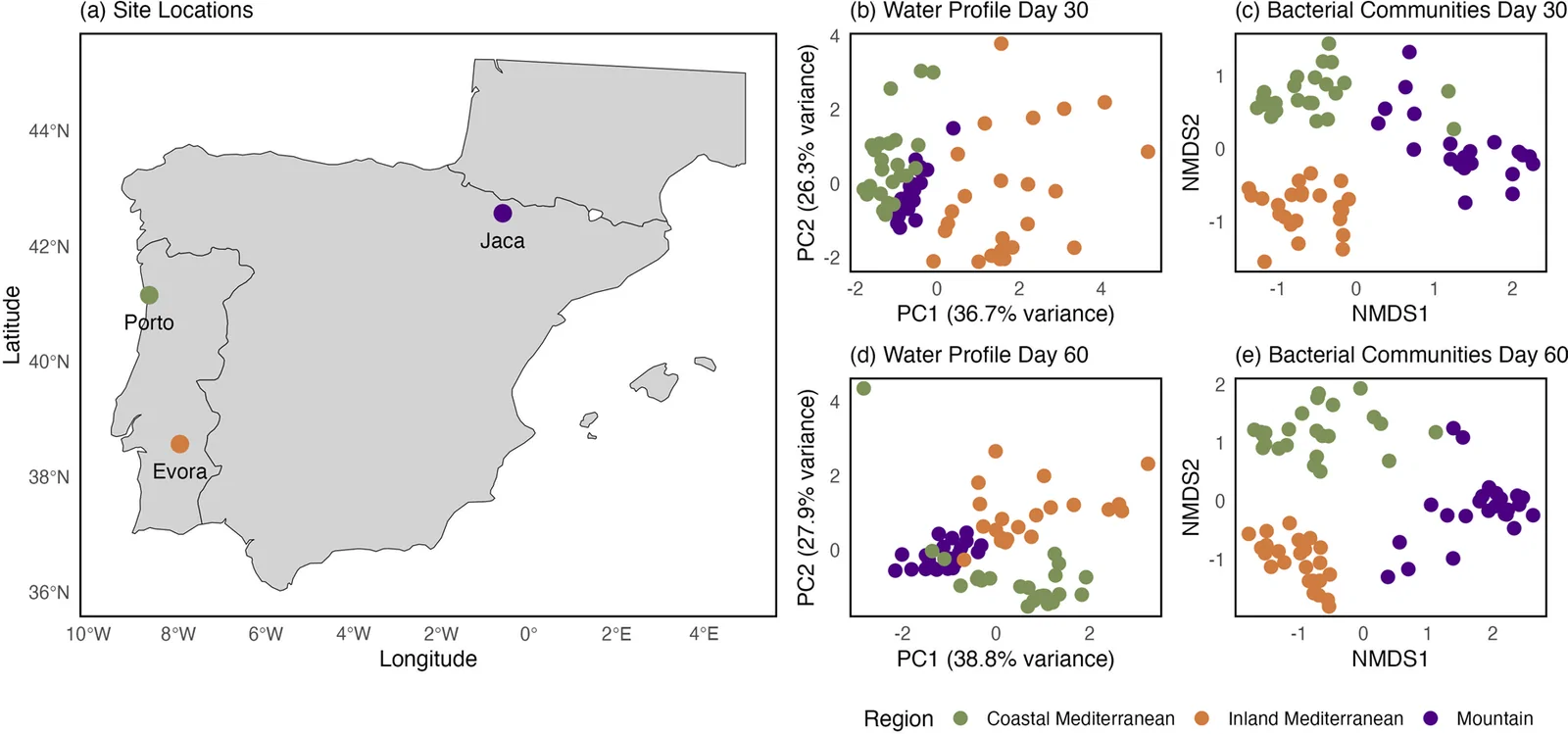
Environmental variables at three regions: mountain (purple), coastal Mediterranean (green) and inland Mediterranean (orange). (a) Map of the Iberian Peninsula denoting the three regions. (b) PCA ordination on normalized biotic and abiotic water variables (dissolved oxygen concentration, pH, conductivity, chlorophyll *a* concentration, nitrate concentration and phosphate concentration) in the three regions 30 days after the start of the experiment (*n* = 24). (c) nMDS ordination on Bray–Curtis dissimilarities calculated on normalized, square-root transformed relative abundances of ASVs in benthic freshwater bacterial communities from three regions 30 days after the start of the experiment (stress = 0.17) (*n* = 24). (d) PCA ordination on normalized biotic and abiotic water variables 60 days after the start of the experiment (dissolved oxygen concentration, pH, conductivity and chlorophyll *a* concentration) (*n* = 24). (e) nMDS ordination on Bray–Curtis dissimilarities calculated on square root transformed relative abundances of ASVs in benthic freshwater bacterial communities 60 days after the start of the experiment (stress = 0.18) (*n* = 24).

### (b) Bacterial community structures differed among regions and salinity treatments, but not nutrients

Bacterial community structure differed significantly among all regions at both time points and for all salinity levels despite interactions with salinity treatment and day (salinity × region × day interaction term: pseudo-*F*_4,50_ = 1.528, *p* = 0.001; figure 2; supplementary material, figure S1 and table S5). Salinity significantly affected bacterial community structure in all regions, although the salinity levels that differed from each other varied across the regions and through time. At day 30, the structure of high salinity and ambient salinity communities differed significantly in all regions; moderate versus high salinity communities differed significantly in the inland and coastal regions but not the mountain region, and ambient versus moderate salinity communities differed significantly only in the coastal region (salinity × region × day interaction term: pseudo-*F*_4,50_ = 1.528, *p* < 0.001; figure 2; supplementary material, table S5). By day 60 all salinity levels differed significantly from each other in all regions (salinity × region × day interaction term: pseudo-*F*_4,50_ = 1.528, *p* < 0.001; figure 2; supplementary material, table S5). Nutrient treatment had no significant effects on bacterial community structure at any region or time (figure 2; supplementary material, table S5).

**Figure 2.**
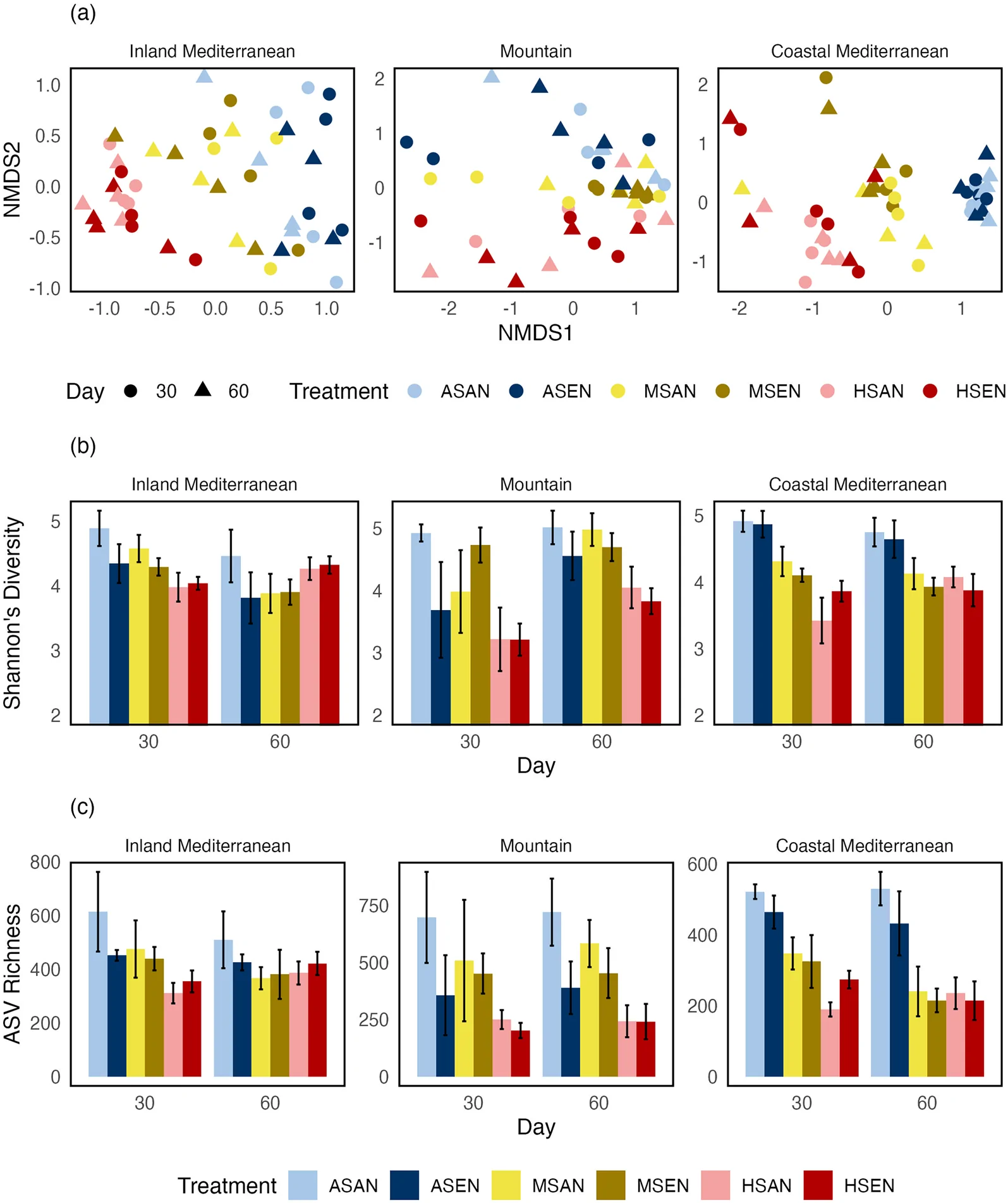
Effects of nutrient enrichment and salinity on bacterial community structure and diversity in benthic freshwater bacterial communities under ambient salinity (AS; light blue), ambient salinity + enriched nutrients (ASEN; dark blue), moderate salinity (MS; yellow), moderate salinity + enriched nutrients (MSEN; brown), high salinity (HS; pink) or high salinity + enriched nutrients (HSEN; red) in three regions (inland Mediterranean, mountain or coastal Mediterranean) after 30 or 60 days. (a) nMDS ordination on Bray–Curtis distances calculated on square root transformed relative ASV abundances (inland stress = 0.15, mountain stress = 0.11 and coastal stress = 0.10). (b) Mean Shannon's diversity of ASVs (±SE) (*n* = 4). (c) Mean ASV richness (±SE) (*n* = 4). Relative abundances of ASVs grouped by phylum among treatments for each location and time point can be seen in figures S3 and S4 in the supplementary material.

Across regions and time, bacterial richness was significantly lower in high salinity communities compared to ambient and moderate salinity communities, and moderate salinity communities had lower richness than ambient salinity communities (*F*_2,54_ = 14.81, *p* < 0.001; figure 2; supplementary material, table S6). Bacterial richness was also significantly lower in the coastal region compared to the mountain and inland regions (*F*_2,53.9_ = 3.31, *p* = 0.044; figure 2; supplementary material, table S6). Shannon diversity in bacterial communities was significantly influenced by the interaction between salinity and region (*F*_4,54_ = 3.19, *p* = 0.020), and the interaction between salinity and day (*F*_2,52_ = 3.97, *p* = 0.025; figure 2; supplementary material, table S6). Shannon's diversity was significantly lower in high salinity communities compared to ambient and moderate salinity communities in the mountain region (salinity × region interaction term: *F*_4,54_ = 3.19, *p* = 0.020; figure 2; supplementary material, table S6). In the coastal region, Shannon's diversity was significantly lower in high and moderate salinity communities compared to ambient salinity communities (salinity × region interaction term: *F*_4,54_ = 3.19, *p* = 0.020; figure 2; supplementary material, table S6). Independent of region, Shannon's diversity was also significantly lower in high salinity communities compared to both moderate and ambient salinity communities at day 30, and lower than ambient at day 60 (salinity × day interaction term: *F*_2,52_ = 3.97, *p* = 0.025; figure 2; supplementary material, table S6). Nutrient treatment had no significant effect on bacterial richness or diversity at any time or region (figure 2; supplementary material, table S6).

### (c) Abundances of phototrophic groups were affected by nutrient enrichment and salinity elevation

Thirty days post nutrient enrichment, benthic biofilm communities had higher abundances of green algae compared to those without nutrient enrichment across all regions (*F*_1,56_ = 4.606, *p* = 0.036), but this effect was no longer detected after 60 days (supplementary material, figure S5 and table S7). After 30 days of treatment, abundances of diatoms and cyanobacteria were higher under high salinity compared to moderate and ambient salinity in the inland region (diatoms: *F*_4,56_ = 3.344, *p* = 0.036; cyanobacteria: *F*_4,54_ = 2.618, *p* = 0.045; supplementary material, figure S5 and table S7). The effects of salinity on these phototrophic groups were also no longer detected after 60 days (supplementary material, figure S5 and table S7).

### (d) Treatment effects on microbial community function varied by region

Community biomass, GPP and CR rates were all significantly higher in high salinity communities compared to ambient and moderate salinity communities after 30 days in the inland region, but NPP was unaffected (salinity × region × day interaction terms: biomass: *F*_2,53_ = 3.536, *p* = 0.012; GPP: *F*_3,46_ = 3.689, *p* = 0.018; CR: *F*_3,46_ = 4.558, *p* = 0.007; figure 3; supplementary material, figure S7 and table S8). After 60 days, both high and moderate salinity communities showed higher GPP and NPP than ambient salinity communities at the inland region (salinity × region × day interaction terms: GPP *F*_3,46_ = 3.689, *p* = 0.018; NPP: *F*_3,46_ = 3.973, *p* = 0.013; figure 3; supplementary material, figure S8 and table S8), while biomass and CR no longer differed (supplementary material, figure S6 and table S8). The opposite effect was seen in the coastal region, where at 60 days ambient salinity communities had significantly higher GPP and NPP than moderate and high salinity communities (salinity × region × day interaction terms: GPP: *F*_3,46_ = 3.689, *p* = 0.018; NPP: *F*_3,46_ = 3.973, *p* = 0.013; supplementary material, figure S8 and table S8). Moderate salinity communities also had lower CR rates than ambient and high salinity communities (salinity × region × day interaction term: *F*_3,46_ = 4.558, *p* = 0.007; supplementary material, figure S8 and table S8).

**Figure 3.**
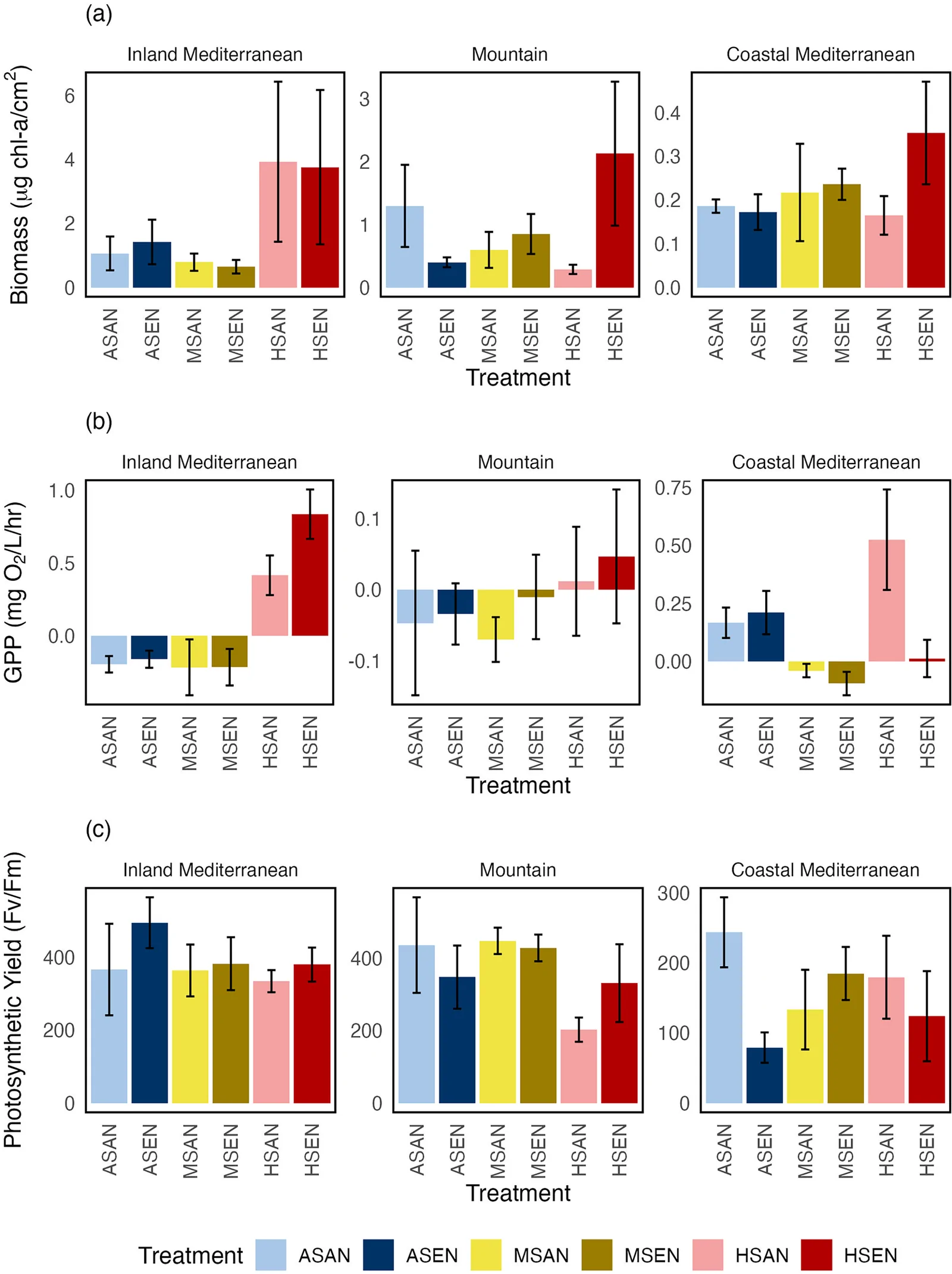
Functions of benthic freshwater bacterial communities under ambient salinity + ambient nutrients (ASAN; light blue), ambient salinity + enriched nutrients (ASEN; dark blue), moderate salinity + ambient nutrient (MSAN; yellow), moderate salinity + enriched nutrients (MSEN; brown), high salinity + ambient nutrients (HSAN; pink) or high salinity + enriched nutrients (HSEN; dark pink) in three regions (inland Mediterranean, mountain or coastal Mediterranean) after 30 days of treatment. (a) Mean biomass in benthic microbial communities (±SE) (*n* = 4). (b) Mean GPP of benthic microbial communities (±SE) (*n* = 4). (c) Mean photosynthetic yield in benthic microbial communities (±SE) (*n* = 4) (data from 60 days are shown in the supplementary material, figures S6 and S8).

There were no significant effects of salinity or nutrient treatments on photosynthetic yields, although they were lower in the coastal region compared to the inland and mountain regions (*F*_2,54_ = 16.14, *p* < 0.001; figure 3; supplementary material, figure S6 and table S8). There were no significant effects of nutrient treatment on biomass, GPP, NPP, CR or photosynthetic yields at any region or time point (figure 3; supplementary material, figure S6 and S7 and table S8).

## 4. Discussion

Our results demonstrate that salinization is a strong and consistent driver of freshwater benthic bacterial community structure across contrasting climatic regions. Bacterial richness and Shannon's diversity declined with stepwise losses in richness and diversity at moderate (+5 g l⁻¹ NaCl) and high (+15 g l⁻¹ NaCl) salinity concentrations compared to ambient. Bacterial communities had distinct structures at each salinity level (ambient, moderate and high), and the differences between these structures increased over time. These strong salinity effects were consistent across coastal Mediterranean, inland Mediterranean and mountain regions that differed in baseline bacterial community structures, productivity and background water chemistry. This highlights the widespread strength of salinity as a global environmental filter of freshwater bacterial diversity and community structures. By contrast to the consistent salinity effects on community structure, functional responses of benthic microbial communities and responses of phototrophic groups varied across regions. There were no significant main or interactive effects of nutrient enrichment on biofilm bacterial community structure or whole-biofilm functioning. Nutrient enrichment increased abundances of green algae in benthic biofilms after 30 days, but nutrient effects were otherwise weaker than expected.

### (a) Salinity as the dominant stressor with consistent structural effects for bacterial communities but variable effects on phototrophic groups and whole-biofilm functions

Salinity emerged as the dominant stressor in all regions. Salinity effects were stronger at high salinity levels compared to moderate salinity levels. This is consistent with the concentration-dependent effects of salinity observed in other freshwater microbial, algal and macroinvertebrate communities [13,68–70]. Although the structural responses of benthic bacterial communities to salinity were consistent across regions, these taxonomic changes did not produce consistent changes in integrated biofilm functioning. This supports previous research demonstrating a mismatch of structural and functional responses to freshwater salinization and nutrient enrichment in freshwater benthic biofilms [12,15]. One possible explanation for this may be that differences in local species pools may have led to different levels of functional redundancy across regions [71]. For example, the mountain biofilm communities had significantly higher baseline bacterial richness compared to those in the coastal region. It is possible that the greater bacterial richness in the mountain region provided greater potential for functional redundancy, maintaining functional rates despite some species being lost [24]. This could explain why GPP decreased under high salinity in the coastal region, but not the mountain region, despite decreases in bacterial richness in both. Another possible explanation is that functional changes corresponded with structural changes in non-bacterial phototrophic communities within the biofilm in some regions. For example, in the inland region, high salinity increased GPP, NPP and abundances of diatoms and cyanobacteria. This suggests a potential link between salinity driven increases in phototrophs and increased rates of productivity.

### (b) Regional context shaped functional outcomes of salinization for biofilms

The effects of salinity treatments on rates of NPP, GPP, CR, biomass and abundances of phototrophic groups showed divergent patterns across regions, highlighting the spatial variability of salinity effects on biofilm functioning. This builds on recent findings that multiple stressor effects vary with the temporal context of stressor exposure [15]. Various mechanisms may explain the variability in functional effects of salinization in biofilms among regions. Firstly, variable functional effects may have been mediated by differences in the way salinity impacted the biotic and abiotic water conditions across regions. For example, high salinity increased the pH of water in the coastal region, but not in the inland or mountain region. Previous research has shown that pH can mediate the effects of salinity for some organisms [72]. High salinity also increased chlorophyll *a* concentrations in water in the inland region, but decreased them in the coastal region, which may have influenced functional changes in the biofilm. Secondly, although not measured here, it is possible that high salinity may have caused regionally specific differences in community structures of unmeasured algal groups or in community structures of higher trophic levels (e.g. grazers). Changes in cross-trophic interactions could have, in turn, affected benthic biofilm biomass and productivity differently across regions.

Undertaking manipulative experiments specifically designed to disentangle the possible drivers of this spatial variation will be a pivotal next step in multiple stressor research. These experiments should independently manipulate possible drivers, for example, environmental conditions, biofilm species pools and community structures in higher trophic levels. Ideally, these experiments should also be replicated across space to identify the factors that predict variation in stressor effects among regions. This would allow identification of particular environmental or community traits that increase ecosystem vulnerability or resilience to multiple stressors. This may allow policymakers to target regions or sites that are most vulnerable, or most likely to benefit from intervention, depending on management goals.

### (c) Limited detectable main or interactive effects of nutrient enrichment

In this study, the only component of the benthic community that was affected by nutrient enrichment was the abundance of green algae, which was higher in nutrient enriched communities compared to ambient after 30 days. Contrary to our predictions, biofilm communities did not detectably shift in structure or function under nutrient enrichment, and there was no evidence of interactive stressor effects. This contrasts with recent findings that nutrient enrichment and freshwater salinization have combined effects on benthic biofilm metabolism that are stronger than individual stressor effects [12,15]. This difference may be owing to differences in nutrient pulse timing between experiments (30 days after the biofilms began developing versus the beginning of biofilm development). The findings of this experiment instead support the findings of a recent meta-analysis showing stressor dominance was a common outcome of multiple stressor scenarios in freshwater ecosystems [2]. Interestingly, in this meta-analysis, nutrient enrichment consistently emerged as the dominant freshwater stressor [2], a trend which is not supported by the results presented here. Another possible explanation for the weak nutrient effects in this experiment is that our systems were highly nutrient limited. Nutrient enrichment in nutrient-limited systems can cause rapid increases in biofilm productivity, benthic chlorophyll *a* concentrations and enzyme activity within days, but these effects often dissipate as resources are depleted or recycled [40,41,73]. Our sampling design (30- and 60-day post-stressor additions) aimed to capture stressor effects that persisted over longer time periods and may have missed possible transient responses. While our results indicate that a single nutrient pulse did not affect bacterial community structure or whole-biofilm function over weeks to months, green algal abundance responded after 30 days, suggesting that phototrophic components may provide a more sensitive indicator of nutrient pulses at these timescales.

Collectively, our findings reveal a potential dichotomy in freshwater benthic biofilm responses to salinization: structural responses in bacterial communities can be generalizable and predictable, whereas phototrophic abundances and whole-biofilm functional outcomes can be context-dependent. The consistent dose-dependent loss of bacterial diversity across regions underscores salinity as a global environmental filter whose structural effects are robust across climates, species pools and baseline environmental conditions. By contrast, the variability of functional responses underscores the importance of regional context, such as species pool composition and environmental conditions in mediating ecosystem responses to salinization. For global change biology and freshwater management, this implies that salinity thresholds may provide globally applicable benchmarks for bacterial diversity conservation. However, tailoring functional targets to regional contexts, for example by targeting systems with lower baseline diversity or certain environmental conditions, may be needed to manage and conserve biofilm function. Recognizing that salinity-driven bacterial diversity loss is consistent, while responses of phototrophic groups and whole-biofilm functional consequences are variable, and will enable more targeted, adaptive management of freshwater ecosystems under accelerating global change.

## Supplementary Material



## Data Availability

Data available in the Dryad repository [74]. Supplementary material is available online [75].
