# Supplementary material for: Consistent diversity loss but divergent functional responses of freshwater biofilms to salinity and nutrient enrichment across climatic regions
Source: Proc Biol Sci. 2026 Oct 21;293(2081):20261161. doi: 10.1098/rspb.2026.1161 (PMC13682921; doi:10.1098/rspb.2026.1161)
Supplement: Supplementary file 1 [file rspb.2026.1161.zip › rspb.2026.1161/rspb20261161_si_001.docx]

Supplementary material

**Title:** Consistent diversity loss but divergent functional responses of freshwater biofilms to salinity and nutrient enrichment across climatic regions.

**Authors**: Rose Fuggle, Mariana Mayer-Pinto, Miguel G. Matias, Ezequiel M. Marzinelli

**Journal**: Proceedings of the Royal Society B

**DOI**: 0.1098/rspb

Figure S1: Environmental variables in experimental pond water under ambient salinity + ambient nutrients (ASAN; light blue), ambient salinity + enriched nutrients (ASEN; dark blue), moderate salinity + ambient nutrient (MSAN; yellow), moderate salinity + enriched nutrients (MSEN; brown), high salinity + ambient nutrients (HSAN; pink) or high salinity + enriched nutrients (HSEN; dark pink) in three regions (inland Mediterranean, mountain or coastal Mediterranean) 30 or 60 days after the start of the experiment. (a) Mean Conductivity. (b) Mean pH. (c) Mean Dissolved Oxygen. (d) Mean concentration of chlorophyll *a.* Data are means (±SE) (n = 4). Note range differences in y-axes among regions.

Figure S2: Nitrate and phosphate concentrations in experimental pond water ambient salinity + ambient nutrients (ASAN; light blue), ambient salinity + enriched nutrients (ASEN; dark blue), moderate salinity + ambient nutrient (MSAN; yellow), moderate salinity + enriched nutrients (MSEN; brown), high salinity + ambient nutrients (HSAN; pink) or high salinity + enriched nutrients (HSEN; dark pink) in three regions (inland Mediterranean, mountain or coastal Mediterranean) 30 day after the start of the experiment. (a) Mean nitrate concentration. (b) Mean phosphate concentration. Data are means (±SE) (n = 4). Note range differences in y-axes among regions.

Figure S3: Mean relative abundances of bacterial ASVs grouped by phylum in freshwater benthic biofilms under ambient salinity + ambient nutrients (ASAN), ambient salinity + enriched nutrients (ASEN), moderate salinity + ambient nutrient (MSAN), moderate salinity + enriched nutrients (MSEN), high salinity + ambient nutrients (HSAN) or high salinity + enriched nutrients (HSEN) in three regions (inland Mediterranean, mountain or coastal Mediterranean) 30 days after the start of the experiment (*n* = 4).

Figure S4: Mean relative abundances of bacterial ASVs grouped by phylum in freshwater benthic biofilms under ambient salinity + ambient nutrients (ASAN), ambient salinity + enriched nutrients (ASEN), moderate salinity + ambient nutrient (MSAN), moderate salinity + enriched nutrients (MSEN), high salinity + ambient nutrients (HSAN) or high salinity + enriched nutrients (HSEN) in three regions (inland Mediterranean, mountain or coastal Mediterranean) 60 days after the start of the experiment (*n* = 4).

Figure S5: Freshwater benthic biofilm abundances of green algae (a), diatoms (b) and cyanobacteria (c) under ambient salinity + ambient nutrients (ASAN; light blue), ambient salinity + enriched nutrients (ASEN; dark blue), moderate salinity + ambient nutrient (MSAN; yellow), moderate salinity + enriched nutrients (MSEN; brown), high salinity + ambient nutrients (HSAN; pink) or high salinity + enriched nutrients (HSEN; dark pink) in three regions (inland Mediterranean, mountain or coastal Mediterranean) 30 days or 60 days after the start of the experiment. Data are means (±SE) (n = 4). Note range differences in y-axes among regions.

Figure S6: Freshwater bacterial community biomass (a) and photosynthetic yield (b) under ambient salinity + ambient nutrients (ASAN; light blue), ambient salinity + enriched nutrients (ASEN; dark blue), moderate salinity + ambient nutrient (MSAN; yellow), moderate salinity + enriched nutrients (MSEN; brown), high salinity + ambient nutrients (HSAN; pink) or high salinity + enriched nutrients (HSEN; dark pink) in three regions (inland Mediterranean, mountain or coastal Mediterranean) 60 days after the start of the experiment. Data are means (±SE) (n = 4). Note range differences in y-axes among regions.

Figure S7: Rates of Net Primary Productivity (NPP) and Community Respiration (CR) of benthic freshwater bacterial communities under ambient salinity + ambient nutrients (ASAN; light blue), ambient salinity + enriched nutrients (ASEN; dark blue), moderate salinity + ambient nutrient (MSAN; yellow), moderate salinity + enriched nutrients (MSEN; brown), high salinity + ambient nutrients (HSAN; pink) or high salinity + enriched nutrients (HSEN; dark pink) in three regions (inland Mediterranean, mountain or coastal Mediterranean) 30 days after the start of the experiment. Data are means (±SE) (n = 4). Note range differences in y-axes among regions.

Figure S8: Rates of Gross Primary Productivity (GPP), Net Primary Productivity (NPP) and Community Respiration (CR) of benthic freshwater bacterial communities under ambient salinity + ambient nutrients (ASAN; light blue), ambient salinity + enriched nutrients (ASEN; dark blue), moderate salinity + ambient nutrient (MSAN; yellow), moderate salinity + enriched nutrients (MSEN; brown), high salinity + ambient nutrients (HSAN; pink) or high salinity + enriched nutrients (HSEN; dark pink) in two regions (inland Mediterranean or coastal Mediterranean) 60 days after the start of the experiment. Data are means (±SE) (n = 4). GPP data was not collected in the mountain region on Day 60 due to equipment failure. Note range differences in y-axes among regions.

Figure S9: PCA ordination on normalised environmental water profiles (Day 30: dissolved oxygen concentration, pH, conductivity, chlorophyll *a* concentration, nitrate concentration, phosphate concentration; Day 60: dissolved oxygen concentration, pH, conductivity, chlorophyll a concentration) in the inland, mountain and coastal regions after 30 days (a) – (c) or 60 days (d) – (f) of treatment with Ambient Salinity + Ambient Nutrients (ASAN; light blue), Ambient Salinity + Enriched Nutrients (ASEN; dark blue), Moderate Salinity + Ambient Nutrients (MSAN; yellow), Moderate Salinity + Enriched Nutrients (MSEN; brown), High Salinity + Ambient Nutrients (HSAN; pink) or High Salinity + Enriched Nutrients (HSEN; red), (*n* = 4). Mean and standard errors of the constituent environmental variables (dissolved oxygen concentration, pH, conductivity, chlorophyll *a* concentration, nitrate concentration, phosphate concentration) among treatments, region and time periods can be seen in Figures S1 and S2.

Table S1: Analysis on the effects of Salinity, Nutrients, Region and Day on conductivity, dissolved oxygen concentration, chlorophyll *a* concentration and pH in experimental freshwater pond water. Salinity was a fixed factor with three levels (Ambient, AS; Moderate, MS; High, HS), Nutrients was a crossed, fixed factor with two levels (Ambient, AN; Enriched, EN), Region was a crossed fixed factor with three levels (Inland Mediterranean, I; Coastal Mediterranean, C; Mountain, M) and Day was a crossed fixed factor with two levels (D30, D60). Pond was a random factor nested in the interaction between Salinity, Nutrients and Region. *p*-values were calculated using the *F*-statistic with Satterthwaite degrees of freedom. Post-hoc contrasts were calculated using estimated marginal means. Only outcomes of significant contrasts are shown (alpha = 0.05).

| Conductivity | *F* | df | df residuals | *p* |
| --- | --- | --- | --- | --- |
| Salinity | 186.579 | 2 | 53.593 | **<0.001** |
| Nutrients | 0.007 | 1 | 53.655 | 0.934 |
| Region | 40.769 | 2 | 53.625 | **<0.001** |
| Day | 83.635 | 1 | 52.801 | **<0.001** |
| Salinity:Nutrients | 0.028 | 2 | 53.677 | 0.972 |
| Salinity:Region | 18.172 | 4 | 53.655 | **<0.001** |
| Nutrients:Region | 0.02 | 2 | 53.701 | 0.980 |
| Salinity:Day | 35.795 | 2 | 52.849 | **<0.001** |
| Nutrients:Day | 0.122 | 1 | 52.931 | 0.728 |
| Region:Day | 37.685 | 2 | 52.897 | **<0.001** |
| Salinity:Nutrients:Region | 0.163 | 4 | 53.72 | 0.956 |
| Salinity:Nutrients:Day | 0.044 | 2 | 52.963 | 0.957 |
| Salinity:Region:Day | 18.496 | 4 | 52.94 | **<0.001** |
| Nutrients:Region:Day | 0.237 | 2 | 53.001 | 0.790 |
| Salinity:Nutrients:Region:Day | 0.063 | 4 | 53.031 | 0.992 |
| Contrasts: Salinity:Region:Day (for levels of Salinity) | | df | *t* | *p* |
| For Region = I and Day = D30 | | df | *t* | *p* |
| AS < HS | | 95.325 | 7.967 | **<0.001** |
| MS < HS | | 95.325 | 5.636 | **<0.001** |
| AS < MS | | 95.325 | 2.330 | **0.022** |
| For Region = M and Day = D30 | | df | *t* | *p* |
| AS < HS | | 95.325 | 4.893 | **<0.001** |
| MS < HS | | 95.325 | 3.304 | **0.001** |
| For Region = C and Day = D30 | | df | *t* | *p* |
| AS < HS | | 95.325 | 6.240 | **<0.001** |
| MS < HS | | 95.325 | 3.914 | **<0.001** |
| For Region = C and Day = D30 | | df | *t* | *p* |
| AS < MS | | 95.325 | 2.326 | **0.022** |
| For Region = I and Day = 60 | | df | *t* | *p* |
| MS < HS | | 97.894 | 15.098 | **<0.001** |
| AS < MS | | 97.894 | 5.643 | **<0.001** |
| For Region = M and Day = 60 | | df | *t* | *p* |
| AS < HS | | 95.325 | 5.331 | **<0.001** |
| MS < HS | | 95.325 | 3.599 | **0.001** |
| For Region = C and Day = 60 | | df | *t* | *p* |
| AS < HS | | 95.325 | 9.608 | **<0.001** |
| MS < HS | | 95.325 | 5.763 | **<0.001** |
| AS < MS | | 95.325 | 3.844 | **<0.001** |
| pH | *F* | df | df residuals | *p* |
| Salinity | 15.741 | 2 | 53.806 | **<0.001** |
| Nutrients | 0.351 | 1 | 53.848 | 0.556 |
| Region | 283.684 | 2 | 53.826 | **<0.001** |
| Day | 151.314 | 1 | 52.43 | **<0.001** |
| Salinity:Nutrients | 0.314 | 2 | 53.862 | 0.732 |
| Salinity:Region | 6.519 | 4 | 53.844 | **<0.001** |
| Nutrients:Region | 0.524 | 2 | 53.876 | 0.595 |
| Salinity:Day | 7.684 | 2 | 52.466 | **0.001** |
| Nutrients:Day | 1.773 | 1 | 52.527 | 0.189 |
| Region:Day | 18.337 | 2 | 52.502 | **<0.001** |
| Salinity:Nutrients:Region | 0.07 | 4 | 53.887 | 0.991 |
| Salinity:Nutrients:Day | 0.805 | 2 | 52.551 | 0.453 |
| Salinity:Region:Day | 5.544 | 4 | 52.535 | **0.001** |
| Nutrients:Region:Day | 1.16 | 2 | 52.582 | 0.321 |
| Salinity:Nutrients:Region:Day | 1.101 | 4 | 52.606 | 0.366 |
| Contrasts: Salinity:Region:Day | | df | *t* | *p* |
| For Site = M and Day = D30 | | df | *t* | *p* |
| HS < AS | | 76.179 | 2.266 | **0.026** |
| For Site = C and Day = D30 | | df | *t* | *p* |
| HS < AS | | 76.179 | 4.722 | **<0.001** |
| MS < AS | | 76.179 | 3.571 | **0.001** |
| For Site = M and Day = D60 | | df | *t* | *p* |
| HS < AS | | 76.179 | 2.557 | **0.013** |
| For Site = C and Day = D60 | | df | *t* | *p* |
| HS < AS | | 76.179 | 6.874 | **<0.001** |
| MS < AS | | 76.179 | 7.178 | **<0.001** |
| Chlorophyll *a* | *F* | df | df residuals | *p* |
| Salinity | 1.110 | 2 | 53.37 | 0.337 |
| Nutrients | 2.439 | 1 | 53.44 | 0.124 |
| Region | 16.933 | 2 | 53.409 | **<0.001** |
| Day | 0.099 | 1 | 53.122 | 0.755 |
| Salinity:Nutrients | 0.448 | 2 | 53.465 | 0.641 |
| Salinity:Region | 3.689 | 4 | 53.444 | **0.010** |
| Nutrients:Region | 0.938 | 2 | 53.495 | 0.398 |
| Salinity:Day | 2.311 | 2 | 53.169 | 0.109 |
| Nutrients:Day | 1.052 | 1 | 53.251 | 0.310 |
| Region:Day | 4.714 | 2 | 53.217 | **0.013** |
| Salinity:Nutrients:Region | 1.448 | 4 | 53.519 | 0.231 |
| Salinity:Nutrients:Day | 1.783 | 2 | 53.281 | 0.178 |
| Salinity:Region:Day | 1.812 | 4 | 53.258 | 0.140 |
| Nutrients:Region:Day | 3.663 | 2 | 53.317 | **0.032** |
| Salinity:Nutrients:Region:Day | 1.126 | 4 | 53.345 | 0.354 |
| Contrasts: Salinity:Region | | df | *t* | *p* |
| AS < HS I | | 56.823 | 3.023 | **0.004** |
| MS < AS C | | 52.583 | 2.357 | **0.022** |
| Contrasts: Nutrients:Region:Day | | df | *t* | *p* |
| AN < EN I D30 | | 105.271 | 2.781 | **0.006** |
| Dissolved Oxygen Concentration | *F* | df | df residuals | *p* |
| Salinity | 0.062 | 2 | 53.395 | 0.940 |
| Nutrients | 0.42 | 1 | 53.464 | 0.520 |
| Region | 27.95 | 2 | 53.433 | **<0.001** |
| Day | 31.592 | 1 | 53.089 | **<0.001** |
| Salinity:Nutrients | 0.13 | 2 | 53.489 | 0.878 |
| Salinity:Region | 3.737 | 4 | 53.468 | **0.009** |
| Nutrients:Region | 1.285 | 2 | 53.518 | 0.285 |
| Salinity:Day | 6.007 | 2 | 53.137 | **0.004** |
| Nutrients:Day | 0.021 | 1 | 53.219 | 0.885 |
| Region:Day | 7.399 | 2 | 53.185 | **0.001** |
| Salinity:Nutrients:Region | 0.354 | 4 | 53.542 | 0.840 |
| Salinity:Nutrients:Day | 0.244 | 2 | 53.25 | 0.785 |
| Salinity:Region:Day | 1.863 | 4 | 53.227 | 0.131 |
| Nutrients:Region:Day | 0.141 | 2 | 53.286 | 0.869 |
| Salinity:Nutrients:Region:Day | 0.219 | 4 | 53.315 | 0.927 |
| Contrasts: Salinity:Day | | df | *t* | *p* |
| AS < HS D30 | | 104.745 | 2.455 | **0.016** |
| HS < AS D60 | | 104.998 | 2.200 | **0.030** |
| Contrasts: Salinity:Region | | df | *t* | *p* |
| AS < HS I | | 56.840 | 2.825 | **0.007** |
| AS < MS I | | 54.762 | 2.047 | **0.045** |
| MS < AS C | | 52.612 | 2.058 | **0.045** |

Table S2: Analysis on the effects of Salinity, Nutrients and Region on nitrate concentration and phosphate concentration in water in freshwater experimental ponds 30 days after the start of the experiment. Salinity was a fixed factor with three levels (Ambient, AS; Moderate, MS; High, HS), Nutrients was a crossed, fixed factor with two levels (Ambient, AN; Enriched, EN) and Region was a crossed fixed factor with three levels (Inland Mediterranean, I; Coastal Mediterranean, C; Mountain, M). *p*-values were calculated using the *F*-statistic with Satterthwaite degrees of freedom. Post-hoc contrasts were calculated using estimated marginal means. Only outcomes of significant contrasts are shown (alpha = 0.05).

| Nitrate Concentration | SS | df | *F* | *p* |
| --- | --- | --- | --- | --- |
| Salinity | 0.047 | 2 | 1.44 | 0.246 |
| Nutrients | 0.231 | 1 | 14.229 | **<0.001** |
| Region | 0.090 | 2 | 2.785 | 0.071 |
| Salinity:Nutrients | 0.001 | 2 | 0.035 | 0.965 |
| Salinity:Region | 0.016 | 4 | 0.249 | 0.909 |
| Nutrients:Region | 0.033 | 2 | 1.028 | 0.365 |
| Salinity:Nutrients:Region | 0.018 | 4 | 0.27 | 0.896 |
| Residuals | 0.875 | 54 |  |  |
| Contrasts: Nutrients |  | df | *t* | *p* |
| AN < EN |  | 54 | 3.772 | **<0.001** |
| Phosphate Concentration | SS | df | *F* | *p* |
| Salinity | 0.745 | 2 | 0.068 | 0.934 |
| Nutrients | 0.041 | 1 | 0.007 | 0.932 |
| Region | 539.473 | 2 | 49.378 | **<0.001** |
| Salinity:Nutrients | 0.595 | 2 | 0.054 | 0.947 |
| Salinity:Region | 0.625 | 4 | 0.029 | 0.998 |
| Nutrients:Region | 0.445 | 2 | 0.041 | 0.960 |
| Salinity:Nutrients:Region | 2.196 | 4 | 0.100 | 0.982 |
| Residuals | 294.987 | 54 |  |  |
| Contrasts: Region |  | df | *t* | *p* |
| M < I |  | 54 | 8.534 | **<0.001** |
| C < I |  | 54 | 8.677 | **<0.001** |

Table S3: Multivariate analysis on the effects of Salinity, Nutrients and Region on Euclidean Distances calculated on normalised environmental (biotic and abiotic) water variables (dissolved oxygen concentration, pH, conductivity, chlorophyll a concentration, nitrate concentration, phosphate concentration) from ponds 30 days after the start of the experiment. Salinity was a fixed factor with three levels (Ambient, AS; Moderate, MS; High, HS), Nutrients was a crossed, fixed factor with two levels (Ambient, AN; Enriched, EN), Region was a crossed fixed factor with three levels (Inland Mediterranean, I; Coastal Mediterranean, C; Mountain, M). *p*-values were calculated using unrestricted permutations of raw data. Only outcomes of significant contrasts are shown (alpha = 0.05). Tests for multivariate dispersion (distances to centroids) were done using PERMDISP in PRIMER.

| SOURCE | df | SS | MS | *Pseudo-F* | Perms | *p*(perm) |
| --- | --- | --- | --- | --- | --- | --- |
| Salinity | 2 | 72.179 | 36.089 | 12.46 | 996 | **0.001** |
| Nutrients | 1 | 15.033 | 15.033 | 5.190 | 997 | **0.001** |
| Region | 2 | 148.12 | 74.062 | 25.57 | 999 | **0.001** |
| Salinity x Region | 4 | 20.664 | 5.166 | 1.784 | 998 | **0.044** |
| Salinity x Nutrients | 2 | 1.726 | 0.863 | 0.298 | 999 | 0.977 |
| Nutrients x Region | 2 | 5.985 | 2.992 | 1.033 | 999 | 0.387 |
| Salinity x Nutrients x Region | 4 | 5.884 | 1.471 | 0.508 | 999 | 0.954 |
| Residuals | 54 | 156.41 | 2.896 |  |  |  |
| Total | 71 | 426 |  |  |  |  |
| Contrasts Salinity x Region (for levels of Salinity) | | | | *t* | Perms | *p* |
| M: MS, AS | | | | 1.670 | 999 | **0.022** |
| M: MS, HS | | | | 2.852 | 999 | **0.001** |
| M: AS, HS | | | | 4.630 | 995 | **0.001** |
| C: MS, AS | | | | 1.869 | 998 | **0.019** |
| C: MS, HS | | | | 1.819 | 998 | **0.039** |
| C: AS, HS | | | | 2.716 | 996 | **0.001** |
| I: MS, AS | | | | 1.303 | 999 | 0.192 |
| I: MS, HS | | | | 1.808 | 998 | **0.024** |
| I: AS, HS | | | | 3.000 | 999 | **0.001** |
| Contrasts Salinity x Region (for levels of Region) | | | | *t* | Perms | *p* |
| AS: M, C | | | | 2.628 | 997 | **0.001** |
| AS: M, I | | | | 3.834 | 998 | **0.004** |
| AS: C, I | | | | 3.812 | 998 | **0.001** |
| MS: M, C | | | | 4.148 | 997 | **0.001** |
| MS: M, I | | | | 2.672 | 998 | **0.001** |
| MS: C, I | | | | 3.526 | 998 | **0.001** |
| HS: M, C | | | | 2.421 | 999 | **0.001** |
| HS: M, I | | | | 2.912 | 999 | **0.002** |
| HS: C, I | | | | 2.918 | 998 | **0.002** |
| Dispersions | | | | df | *F* | *p* |
| Salinity | | | | 2, 69 | 5.478 | **0.036** |
| Nutrients | | | | 1, 70 | 0.920 | 0.44 |
| Region | | | | 2, 69 | 25.845 | **0.001** |
| Distance to Centroid for Salinity | | | | Mean | SE |  |
| AS | | | | 1.70 | 0.15 |  |
| MS | | | | 1.86 | 0.16 |  |
| HS | | | | 2.49 | 0.22 |  |
| Distance to Centroid for Region | | | | Mean | SE |  |
| M | | | | 0.99 | 0.10 |  |
| C | | | | 1.67 | 0.14 |  |
| I | | | | 2.50 | 0.19 |  |

Table S4: Multivariate analysis on the effects of Salinity, Nutrients and Region on Euclidean Distances calculated on normalised environmental (biotic and abiotic) water variables (dissolved oxygen concentration, pH, conductivity and chlorophyll a concentration) from ponds 60 days after the start of the experiment. Salinity was a fixed factor with three levels (Ambient, AS; Moderate, MS; High, HS), Nutrients was a crossed, fixed factor with two levels (Ambient, AN; Enriched, EN), Region was a crossed fixed factor with three levels (Inland Mediterranean, I; Coastal Mediterranean, C; Mountain, M). *p*-values were calculated using unrestricted permutations of raw data. Only outcomes of significant contrasts are shown (alpha = 0.05). Tests for multivariate dispersion (distances to centroids) were done using PERMDISP in PRIMER.

| SOURCE | df | SS | MS | *Pseudo-F* | perms | *p*(perm) |
| --- | --- | --- | --- | --- | --- | --- |
| Salinity | 2 | 47.431 | 23.716 | 16.615 | 999 | **0.001** |
| Nutrients | 1 | 0.338 | 0.338 | 0.237 | 998 | 0.898 |
| Region | 2 | 114.6 | 57.301 | 40.145 | 999 | **0.001** |
| Salinity x Region | 4 | 30.143 | 7.536 | 5.280 | 996 | **0.001** |
| Salinity x Nutrients | 2 | 2.067 | 1.034 | 0.724 | 999 | 0.625 |
| Nutrients x Region | 2 | 4.207 | 2.103 | 1.474 | 999 | 0.219 |
| Salinity x Nutrients x Region | 4 | 4.228 | 1.057 | 0.740 | 999 | 0.722 |
| Residuals | 52 | 74.222 | 1.427 |  |  |  |
| Total | 69 | 276 |  |  |  |  |
| Contrasts Salinity x Region (for levels of Salinity) | | | | *t* | Perms | *p* |
| M: MS, HS | | | | 2.389 | 999 | **0.007** |
| M: AS, HS | | | | 3.051 | 998 | **0.001** |
| C: MS, AS | | | | 2.393 | 999 | **0.007** |
| C: MS, HS | | | | 2.060 | 999 | **0.014** |
| C: AS, HS | | | | 2.582 | 997 | **0.003** |
| I: MS, AS | | | | 2.160 | 998 | **0.007** |
| I: MS, HS | | | | 3.492 | 998 | **0.001** |
| I: AS, HS | | | | 5.656 | 998 | **0.001** |
| Contrasts Salinity x Region (for levels of Region) | | | | *t* | Perms | *p* |
| AS: M, C | | | | 1.920 | 999 | **0.012** |
| AS: M, I | | | | 6.184 | 999 | **0.001** |
| AS: C, I | | | | 2.272 | 999 | **0.002** |
| MS: M, C | | | | 10.146 | 999 | **0.001** |
| MS: M, I | | | | 4.291 | 997 | **0.001** |
| Contrasts Salinity x Region (for levels of Region) | | | | *t* | Perms | *p* |
| MS: C, I | | | | 5.411 | 998 | **0.001** |
| HS: M, C | | | | 5.885 | 999 | **0.001** |
| HS: M, I | | | | 6.034 | 999 | **0.001** |
| HS: C, I | | | | 4.682 | 999 | **0.001** |
| Dispersions | | | | df | *F* | *p* |
| Salinity | | | | 2, 67 | 0.828 | 0.610 |
| Region | | | | 2, 67 | 6.352 | **0.003** |
| Distance to Centroid for Region | | | | Mean | SE |  |
| M | | | | 0.668 | 0.064 |  |
| C | | | | 1.418 | 0.261 |  |
| I | | | | 1.506 | 0.166 |  |

Table S5: Multivariate analysis on the effects of Salinity, Nutrients, Region and Day on Bray Curtis dissimilarities among sample pairs calculated based on square root transformed relative abundances of ASVs. Salinity was a fixed factor with three levels (Ambient, AS; Moderate, MS; High, HS), Nutrients was a crossed, fixed factor with two levels (Ambient, AN; Enriched, EN), Region was a crossed fixed factor with three levels (Inland Mediterranean, I; Coastal Mediterranean, C; Mountain, M) and Day was a crossed, fixed factor with two levels (D30, D60). Pond was a random factor nested in the interaction between Salinity, Nutrients and Region. *p*-values were calculated using permutations (perms) of residuals under a reduced model. Tests for multivariate dispersion (distances to centroids) were done using PERMDISP in PRIMER.

| Source | df | SS | MS | *Pseudo-F* | *p*(perm) | Perms |
| --- | --- | --- | --- | --- | --- | --- |
| Salinity | 2 | 35277 | 17639 | 3.947 | **0.001** | 997 |
| Nutrients | 1 | 4209 | 4209 | 0.943 | 0.644 | 995 |
| Day | 1 | 4810 | 4810 | 2.494 | **0.001** | 996 |
| Region | 2 | 100550 | 50275 | 11.259 | **0.001** | 996 |
| Salinity × Nutrients | 2 | 8528 | 4264 | 0.954 | 0.664 | 995 |
| Salinity × Day | 2 | 6015 | 3008 | 1.559 | **0.003** | 998 |
| Salinity × Region | 4 | 57544 | 14386 | 3.218 | **0.001** | 995 |
| Nutrients × Day | 1 | 1684 | 1684 | 0.873 | 0.691 | 996 |
| Nutrients × Region | 2 | 8934 | 4467 | 1.000 | 0.478 | 998 |
| Day × Region | 2 | 8581 | 4291 | 2.224 | **0.001** | 998 |
| Salinity × Nutrients × Day | 2 | 3794 | 1897 | 0.984 | 0.537 | 998 |
| Salinity × Nutrients × Region | 4 | 16547 | 4137 | 0.925 | 0.797 | 992 |
| Salinity × Day × Region | 4 | 11789 | 2947 | 1.528 | **0.001** | 996 |
| Nutrients × Day × Region | 2 | 3408 | 1704 | 0.884 | 0.779 | 998 |
| Pond(Region × Salinity × Nutrients) | 55 | 249060 | 4528 | 2.348 | **0.001** | 985 |
| Salinity × Nutrients × Day × Region | 4 | 7162 | 1790 | 0.928 | 0.695 | 995 |
| Res | 50 | 96445 | 1929 |  |  |  |
| Total | 140 | 630730 |  |  |  |  |
| Contrasts Salinity:Day:Region (for levels of Salinity) | | | | *t* | Perms | *p* |
| For Site = I and Day = D30 | | | | *t* | Perms | *p* |
| HS, AS | | | | 2.144 | 998 | **0.001** |
| HS, MS | | | | 1.824 | 999 | **0.001** |
| For Site = M and Day = D30 | | | | *t* | Perms | *p* |
| HS, AS | | | | 1.341 | 999 | **0.020** |
| For Site = C and Day = D30 | | | | *t* | Perms | *p* |
| C D30 HS, AS | | | | 2.527 | 997 | **0.001** |
| C D30 HS, MS | | | | 1.643 | 997 | **0.003** |
| C D30 AS, MS | | | | 2.039 | 998 | **0.001** |
| For Site = I and Day = D60 | | | | *t* | Perms | *p* |
| HS, AS | | | | 2.271 | 998 | **0.002** |
| HS, MS | | | | 1.827 | 999 | **0.001** |
| AS, MS | | | | 1.443 | 999 | **0.011** |
| For Site = M and Day = D60 | | | | *t* | Perms | *p* |
| HS, AS | | | | 1.336 | 998 | **0.029** |
| HS, MS | | | | 1.493 | 998 | **0.010** |
| AS, MS | | | | 1.452 | 999 | **0.014** |
| For Site = C and Day = D60 | | | | *t* | Perms | *p* |
| HS, AS | | | | 2.218 | 996 | **0.001** |
| HS, MS | | | | 1.227 | 997 | **0.044** |
| AS, MS | | | | 2.039 | 997 | **0.001** |
| Contrasts Salinity:Day:Region (for levels of Region) | | | | *t* | Perms | *p* |
| For Salinity = AS and Day = 30 | | | | *t* | Perms | *p* |
| I, M | | | | 1.747 | 998 | **0.001** |
| I, C | | | | 2.418 | 994 | **0.001** |
| M, C | | | | 2.272 | 997 | **0.001** |
| For Salinity = AS and Day = 60 | | | | *t* | Perms | *p* |
| I, M | | | | 2.023 | 998 | **0.002** |
| I, C | | | | 2.398 | 999 | **0.001** |
| M, C | | | | 2.366 | 996 | **0.002** |
| For Salinity = MS and Day = 30 | | | | *t* | Perms | *p* |
| I, M | | | | 1.979 | 998 | **0.001** |
| I, C | | | | 1.949 | 998 | **0.001** |
| M, C | | | | 2.001 | 999 | **0.002** |
| For Salinity = MS and Day = 60 | | | | *t* | Perms | *p* |
| I, M | | | | 2.628 | 996 | **0.002** |
| I, C | | | | 2.016 | 998 | **0.002** |
| M, C | | | | 2.478 | 998 | **0.001** |
| For Salinity = HS and Day = 30 | | | | *t* | Perms | *p* |
| I, M | | | | 2.187 | 997 | **0.001** |
| I, C | | | | 2.337 | 996 | **0.001** |
| M, C | | | | 1.928 | 998 | **0.002** |
| For Salinity = HS and Day = 60 | | | | *t* | Perms | *p* |
| I, M | | | | 2.099 | 998 | **0.001** |
| I, C | | | | 2.293 | 998 | **0.001** |
| M, C | | | | 1.649 | 999 | **0.002** |
| Dispersions | | | | df | *F* | *p* |
| Salinity | | | | 2, 138 | 0.028 | 0.973 |
| Day | | | | 2, 139 | 0.053 | 0.804 |
| Region | | | | 2, 138 | 0.214 | 0.815 |

Table S6: Analysis on the effects of Salinity, Nutrients, Region and Day on ASV richness and on Shannon’s Diversity calculated on relative abundances of ASVs. Salinity was a fixed factor with three levels (Ambient, AS; Moderate, MS; High, HS), Nutrients was a crossed, fixed factor with two levels (Ambient, AN; Enriched, EN), Region was a crossed fixed factor with three levels (Inland Mediterranean, I; Coastal Mediterranean, C; Mountain, M) and Day was a crossed, fixed factor with two levels (D30, D60). Pond was random factor nested in the interaction between Salinity, Nutrients and Region. *p*-values were calculated using the *F*-statistic with Satterthwaite degrees of freedom. Post-hoc contrasts were calculated using estimated marginal means. Only outcomes of significant contrasts are shown (alpha = 0.05).

| ASV Richness | *F* | df | df residuals | *p* |
| --- | --- | --- | --- | --- |
| Salinity | 14.806 | 2 | 53.909 | **<0.001** |
| Nutrients | 3.931 | 1 | 53.951 | 0.053 |
| Region | 3.310 | 2 | 53.897 | **0.044** |
| Day | 0.277 | 1 | 52.177 | 0.601 |
| Salinity:Nutrients | 2.716 | 2 | 53.978 | 0.075 |
| Salinity:Region | 1.839 | 4 | 53.953 | 0.135 |
| Nutrients:Region | 1.497 | 2 | 53.966 | 0.233 |
| Salinity:Day | 0.783 | 2 | 52.373 | 0.463 |
| Nutrients:Day | 0.002 | 1 | 52.446 | 0.965 |
| Region:Day | 0.693 | 2 | 52.298 | 0.505 |
| Salinity:Nutrients:Region | 0.313 | 4 | 53.994 | 0.868 |
| Salinity Nutrients:Day | 0.058 | 2 | 52.554 | 0.944 |
| Salinity:Region:Day | 0.593 | 4 | 52.465 | 0.670 |
| Nutrients:Region:Day | 0.3063 | 2 | 52.461 | 0.737 |
| Salinity:Nutrients:Region:Day | 0.1212 | 4 | 52.569 | 0.974 |
| Contrasts Salinity |  | df | *t* | *p* |
| HS < AS |  | 52.802 | 5.478 | **<0.001** |
| HS < MS |  | 54.348 | 2.838 | **0.006** |
| MS < AS |  | 55.011 | 2.617 | **0.011** |
| Contrasts Richness |  | df | *t* | ***p*** |
| C < I |  | 53.68 | 2.268 | **0.027** |
| C < M |  | 54.348 | 2.162 | **0.035** |
| Shannon’s Diversity | *F* | df | df residuals | *p* |
| Salinity | 12.987 | 2 | 53.878 | **<0.001** |
| Nutrients | 2.400 | 1 | 53.920 | 0.127 |
| Region | 0.004 | 2 | 53.864 | 0.996 |
| Day | 1.596 | 1 | 52.231 | 0.212 |
| Salinity:Nutrients | 1.882 | 2 | 53.948 | 0.162 |
| Salinity:Region | 3.185 | 4 | 53.922 | **0.020** |
| Nutrients:Region | 0.282 | 2 | 53.934 | 0.755 |
| Salinity:Day | 3.973 | 2 | 52.425 | **0.025** |
| Nutrients:Day | 0.300 | 1 | 52.498 | 0.586 |
| Region:Day | 7.798 | 2 | 52.350 | **0.001** |
| Salinity:Nutrients:Region | 0.726 | 4 | 53.965 | 0.578 |
| Salinity Nutrients:Day | 0.753 | 2 | 52.605 | 0.476 |
| Salinity:Region:Day | 0.445 | 4 | 52.516 | 0.775 |
| Nutrients:Region:Day | 0.268 | 2 | 52.513 | 0.766 |
| Salinity:Nutrients:Region:Day | 1.459 | 4 | 52.619 | 0.228 |
| Contrasts: Salinity:Region |  | df | *t* | *p* |
| HS < AS M |  | 54.085 | 3.962 | **<0.001** |
| HS < MS M |  | 54.085 | 4.094 | **<0.001** |
| HS < AS C |  | 52.083 | 4.034 | **<0.001** |
| MS < AS C |  | 54.827 | 2.827 | **0.007** |
| Contrasts: Salinity:Day |  | df | *t* | *p* |
| HS < AS D30 |  | 97.642 | 5.594 | **<0.001** |
| HS < MS D30 |  | 98.739 | 3.977 | **<0.001** |
| HS < AS D60 |  | 96.880 | 2.683 | **0.009** |

Table S7: Analysis on the effects of Salinity, Nutrients, Region and Day on abundances of green algae, diatoms and cyanobacteria in benthic microbial communities. Salinity was a fixed factor with three levels (Ambient, AS; Moderate, MS; High, HS), Nutrients was a crossed, fixed factor with two levels (Ambient, AN; Enriched, EN), Region was a crossed fixed factor with three levels (Inland Mediterranean, I; Coastal Mediterranean, C; Mountain, M) and Day was a crossed fixed factor with two levels (D30, D60). Pond was included as a random factor nested in the interaction between Salinity, Nutrients and Region. *p*-values were calculated using the *F*-statistic with Satterthwaite degrees of freedom. Post-hoc contrasts were calculated using estimated marginal means. Only outcomes of significant contrasts are shown (alpha = 0.05).

| Green Algae | *F* | | df | df residuals | *p* |
| --- | --- | --- | --- | --- | --- |
| Salinity | 3.058 | | 2 | 53.839 | 0.055 |
| Nutrients | 1.138 | | 1 | 53.855 | 0.291 |
| Region | 5.99 | | 2 | 53.839 | **0.004** |
| Day | 3.126 | | 1 | 53.354 | 0.083 |
| Salinity:Nutrients | 1.476 | | 2 | 53.874 | 0.238 |
| Salinity:Region | 0.575 | | 4 | 53.865 | 0.682 |
| Nutrients:Region | 1.167 | | 2 | 53.874 | 0.319 |
| Salinity:Day | 3.042 | | 2 | 53.397 | 0.056 |
| Nutrients:Day | 4.606 | | 1 | 53.416 | **0.036** |
| Location:Day | 1.324 | | 2 | 53.397 | 0.275 |
| Salinity:Nutrients:Region | 0.572 | | 4 | 53.89 | 0.684 |
| Salinity:Nutrients:Day | 2.195 | | 2 | 53.446 | 0.121 |
| Salinity:Region:Day | 2.025 | | 4 | 53.436 | 0.104 |
| Nutrients:Region:Day | 1.033 | | 2 | 53.446 | 0.363 |
| Salinity:Nutrients:Region:Day | 1.094 | | 4 | 53.474 | 0.369 |
| Contrasts: Nutrients:Day | | | df | *t* | *p* |
| AN < EN D30 | | | 92.38 | 2.051 | **0.043** |
| Diatoms | | *F* | df | df residuals | *p* |
| Salinity | | 1.711 | 2 | 53.754 | 0.190 |
| Nutrients | | 0.024 | 1 | 53.771 | 0.877 |
| Region | | 11.267 | 2 | 53.754 | **<0.001** |
| Day | | 5.501 | 1 | 53.489 | **0.023** |
| Salinity:Nutrients | | 0.018 | 2 | 53.794 | 0.983 |
| Salinity:Region | | 1.182 | 4 | 53.785 | 0.329 |
| Nutrients:Region | | 0.485 | 2 | 53.794 | 0.618 |
| Salinity:Day | | 5.351 | 2 | 53.534 | **0.008** |
| Nutrients:Day | | 0.253 | 1 | 53.555 | 0.617 |
| Location:Day | | 1.206 | 2 | 53.534 | 0.307 |
| Salinity:Nutrients:Region | | 1.162 | 4 | 53.815 | 0.338 |
| Salinity:Nutrients:Day | | 0.082 | 2 | 53.585 | 0.922 |
| Salinity:Region:Day | | 3.344 | 4 | 53.575 | **0.016** |
| Nutrients:Region:Day | | 0.496 | 2 | 53.585 | 0.612 |
| Salinity:Nutrients:Region:Day | | 0.957 | 4 | 53.613 | 0.439 |
| Contrasts: Salinity:Region:Day | | | df | *t* | *p* |
| AS < HS I D30 | | | 102.95 | 4.293 | *<0.001* |
| MS < HS I D30 | | | 102.95 | 4.647 | *<0.001* |
| Cyanobacteria | | *F* | df | df residuals | *p* |
| Salinity | | 0.619 | 2 | 53.788 | 0.542 |
| Nutrients | | 0.009 | 1 | 53.805 | 0.926 |
| Region | | 4.817 | 2 | 53.788 | **0.012** |
| Day | | 0.02 | 1 | 53.439 | 0.889 |
| Salinity:Nutrients | | 0.586 | 2 | 53.827 | 0.560 |
| Salinity:Region | | 0.371 | 4 | 53.817 | 0.829 |
| Nutrients:Region | | 0.071 | 2 | 53.827 | 0.932 |
| Salinity:Day | | 3.068 | 2 | 53.483 | 0.055 |
| Nutrients:Day | | 0.041 | 1 | 53.504 | 0.840 |
| Location:Day | | 0.395 | 2 | 53.483 | 0.676 |
| Salinity:Nutrients:Region | | 0.364 | 4 | 53.846 | 0.833 |
| Salinity:Nutrients:Day | | 0.423 | 2 | 53.534 | 0.657 |
| Salinity:Region:Day | | 2.618 | 4 | 53.524 | **0.045** |
| Nutrients:Region:Day | | 0.132 | 2 | 53.534 | 0.877 |
| Salinity:Nutrients:Region:Day | | 0.306 | 4 | 53.563 | 0.872 |
| Contrasts: Salinity:Region:Day | | | df | *t* | *p* |
| AS < HS I D30 | | | 99.315 | 2.340 | **0.021** |
| MS < HS I D30 | | | 99.315 | *3.259* | **0.002** |

Table S8: Analysis on the effects of Salinity, Nutrients, Region and Day on biomass, photosynthetic yield, gross primary productivity rate (GPP), net primary productivity rate (NPP) and community respiration rate (CR) in benthic microbial communities. Salinity was a fixed factor with three levels (Ambient, AS; Moderate, MS; High, HS), Nutrients was a crossed, fixed factor with two levels (Ambient, AN; Enriched, EN), Region was a crossed fixed factor with three levels (Inland Mediterranean, I; Coastal Mediterranean, C; Mountain, M) and Day was a crossed fixed factor with two levels (D30, D60). Pond was included as a random factor nested in the interaction between Salinity, Nutrients and Region. *p*-values were calculated using the *F*-statistic with Satterthwaite degrees of freedom. Post-hoc contrasts were calculated using estimated marginal means. Only outcomes of significant contrasts are shown (alpha = 0.05). Note CR, NPP and GPP datasets for the mountain region are incomplete on D60.

| Biomass | *F* | df | df residuals | *p* |
| --- | --- | --- | --- | --- |
| Salinity | 1.132 | 2 | 53.827 | 0.330 |
| Nutrients | 0.048 | 1 | 53.843 | 0.828 |
| Region | 7.106 | 2 | 53.827 | **0.002** |
| Day | 1.63 | 1 | 53.375 | 0.207 |
| Salinity:Nutrients | 0.478 | 2 | 53.863 | 0.623 |
| Salinity:Region | 0.519 | 4 | 53.854 | 0.722 |
| Nutrients:Region | 0.232 | 2 | 53.863 | 0.794 |
| Salinity:Day | 4.828 | 2 | 53.418 | **0.012** |
| Nutrients:Day | 0.31 | 1 | 53.438 | 0.580 |
| Location:Day | 0.895 | 2 | 53.418 | 0.415 |
| Salinity:Nutrients:Region | 0.511 | 4 | 53.88 | 0.728 |
| Salinity:Nutrients:Day | 0.445 | 2 | 53.468 | 0.643 |
| Salinity:Region:Day | 3.536 | 4 | 53.458 | **0.012** |
| Nutrients:Region:Day | 0.118 | 2 | 53.468 | 0.889 |
| Salinity:Nutrients:Region:Day | 0.391 | 4 | 53.496 | 0.814 |
| Contrasts: Salinity:Region:Day | | df | *t* | *p* |
| AS < HS I D30 | | 93.60 | 3.370 | **0.001** |
| MS < HS I D30 | | 93.60 | 4.049 | **<0.001** |
| Photosynthetic Yield | *F* | df | df residuals | *p* |
| Salinity | 1.874 | 2 | 53.662 | 0.163 |
| Nutrients | 0 | 1 | 53.679 | 0.996 |
| Region | 16.14 | 2 | 53.662 | **<0.001** |
| Day | 0.002 | 1 | 53.613 | 0.968 |
| Salinity:Nutrients | 0.598 | 2 | 53.704 | 0.553 |
| Salinity:Region | 0.897 | 4 | 53.695 | 0.473 |
| Nutrients:Region | 2.744 | 2 | 53.704 | 0.073 |
| Salinity:Day | 0.143 | 2 | 53.655 | 0.867 |
| Nutrients:Day | 0.044 | 1 | 53.674 | 0.834 |
| Location:Day | 2.746 | 2 | 53.655 | 0.073 |
| Salinity:Nutrients:Region | 1.58 | 4 | 53.727 | 0.193 |
| Salinity:Nutrients:Day | 1.807 | 2 | 53.701 | 0.174 |
| Salinity:Region:Day | 0.659 | 4 | 53.692 | 0.623 |
| Nutrients:Region:Day | 0.825 | 2 | 53.701 | 0.444 |
| Salinity:Nutrients:Region:Day | 1.13 | 4 | 53.726 | 0.352 |
| Contrasts: Region |  | df | *t* | *p* |
| C < I |  | 53.249 | 4.900 | **<0.001** |
| C < M |  | 53.987 | 4.950 | **<0.001** |
| GPP | *F* | df | df residuals | *p* |
| Salinity | 1.607 | 2 | 54.58 | 0.210 |
| Nutrients | 0.583 | 1 | 52.86 | 0.449 |
| Region | 2.499 | 2 | 55.291 | 0.091 |
| Day | 33.038 | 1 | 45.72 | **<0.001** |
| Salinity:Nutrients | 0.314 | 2 | 57.08 | 0.732 |
| Salinity:Region | 3.632 | 4 | 62.183 | **0.010** |
| Nutrients:Region | 0.319 | 2 | 58.303 | 0.728 |
| Salinity:Day | 0.869 | 2 | 45.831 | 0.426 |
| Nutrients:Day | 0.853 | 1 | 45.439 | 0.361 |
| Location:Day | 2.624 | 2 | 45.96 | 0.083 |
| Salinity:Nutrients:Region | 0.551 | 4 | 69.09 | 0.699 |
| Salinity:Nutrients:Day | 0.304 | 2 | 45.562 | 0.739 |
| Salinity:Region:Day | 3.689 | 3 | 46.192 | **0.018** |
| Nutrients:Region:Day | 1.968 | 2 | 45.268 | 0.152 |
| Salinity:Nutrients:Region:Day | 1.532 | 2 | 45.601 | 0.227 |
| Contrasts: Salinity:Region:Day |  | df | *t* | *p* |
| Region = I and Day = D30 |  | df | *t* | *p* |
| AS < HS |  | 95.760 | 2.055 | **0.043** |
| MS < HS |  | 95.760 | 2.152 | **0.034** |
| Region = I and Day = D60 |  | df | *t* | *p* |
| AS < HS |  | 95.760 | 2.018 | **0.046** |
| AS < MS |  | 96.144 | 2.817 | **0.006** |
| Region = C and Day = D60 |  | df | *t* | *p* |
| HS < AS |  | 95.760 | 2.136 | **0.035** |
| MS < AS |  | 95.760 | 3.963 | **0.001** |
| NPP | *F* | df | df residuals | *p* |
| Salinity | 0.199 | 2 | 54.503 | 0.820 |
| Nutrients | 0.315 | 1 | 52.747 | 0.577 |
| Region | 0.786 | 2 | 55.252 | 0.461 |
| Day | 17.909 | 1 | 45.825 | **<0.001** |
| Salinity:Nutrients | 1.35 | 2 | 57.005 | 0.267 |
| Salinity:Region | 3.151 | 4 | 62.298 | **0.020** |
| Nutrients:Region | 0.224 | 2 | 58.29 | 0.800 |
| Salinity:Day | 0.493 | 2 | 45.937 | 0.614 |
| Nutrients:Day | 0.447 | 1 | 45.54 | 0.507 |
| Location:Day | 1.868 | 2 | 46.066 | 0.166 |
| Salinity:Nutrients:Region | 0.105 | 4 | 69.335 | 0.980 |
| Salinity:Nutrients:Day | 1.432 | 2 | 45.663 | 0.249 |
| Salinity:Region:Day | 3.973 | 3 | 46.299 | **0.013** |
| Nutrients:Region:Day | 1.031 | 2 | 45.367 | 0.365 |
| Salinity:Nutrients:Region:Day | 1.655 | 2 | 45.702 | 0.202 |
| Contrasts: Salinity:Region:Day |  | df | *t* | *p* |
| Region = I and Day = D60 |  | df | *t* | *p* |
| AS < HS |  | 96.134 | 2.114 | **0.037** |
| AS < MS |  | 96.404 | 2.989 | **0.004** |
| Region = C and Day = D60 |  | df | *t* | *P* |
| HS < AS |  | 96.133 | 3.243 | **0.002** |
| MS < AS |  | 96.132 | 3.456 | **0.001** |
| CR | *F* | df | df residuals | *p* |
| Salinity | 6.929 | 2 | 54.532 | **0.002** |
| Nutrients | 0.455 | 1 | 52.789 | 0.503 |
| Region | 3.723 | 2 | 55.267 | **0.030** |
| Day | 23.569 | 1 | 45.787 | **<0.001** |
| Salinity:Nutrients | 1.557 | 2 | 57.033 | 0.220 |
| Salinity:Region | 1.224 | 4 | 62.257 | 0.310 |
| Nutrients:Region | 0.488 | 2 | 58.295 | 0.617 |
| Salinity:Day | 0.69 | 2 | 45.898 | 0.507 |
| Nutrients:Day | 0.643 | 1 | 45.503 | 0.427 |
| Location:Day | 1.722 | 2 | 46.027 | 0.190 |
| Salinity:Nutrients:Region | 1.504 | 4 | 69.246 | 0.210 |
| Salinity:Nutrients:Day | 1.785 | 2 | 45.626 | 0.179 |
| Salinity:Region:Day | 4.558 | 3 | 46.26 | **0.007** |
| Nutrients:Region:Day | 2.358 | 2 | 45.331 | 0.106 |
| Salinity:Nutrients:Region:Day | 0.66 | 2 | 45.665 | 0.522 |
| Contrasts: Salinity:Region:Day |  | df | *t* | *p* |
| Region = I and Day = D30 |  | df | *t* | *p* |
| AS < HS |  | 96.004 | 3.712 | **<0.001** |
| MS < HS |  | 96.004 | 3.812 | **<0.001** |
| Region = C and Day = D60 |  | df | *t* | *P* |
| MS < HS |  | 96.004 | 3.598 | **0.001** |
| MS < AS |  | 96.004 | 2.465 | **0.015** |
